# Can Online Exercise Using Wearable Devices Improve Perceived Well-Being? A Study Among Patients with Coronary Artery Disease

**DOI:** 10.3390/s25030698

**Published:** 2025-01-24

**Authors:** Apostolia Ntovoli, Alexandros Mitropoulos, Maria Anifanti, Georgia Koukouvou, Evangelia Kouidi, Kostas Alexandris

**Affiliations:** 1 Department of Physical Education and Sports Sciences, Frederick University, Nicosia 3080, Cyprus; antovoli@phed.auth.gr; 2 Laboratory of Management of Sports Recreation and Tourism, Department of Physical Education and Sport Science, Aristotle University of Thessaloniki, 57001 Thessaloniki, Greece; 3 Lifestyle, Exercise and Nutritional Improvement (LENI) Research Group, Department of Nursing and Midwifery, Sheffield Hallam University, Sheffield S1 1WB, UK; alexandros.mitropoulos@shu.ac.uk; 4 Laboratory of Sport Medicine, Department of Physical Education and Sport Science, Aristotle University of Thessaloniki, 57001 Thessaloniki, Greece; manyfant@phed.auth.gr (M.A.); gkoukouvou@gmail.com (G.K.); kouidi@phed.auth.gr (E.K.)

**Keywords:** subjective well-being, PERMA, online exercise rehabilitation programs, wearable devices, telerehabilitation, patients with coronary artery disease, online exercise protocol

## Abstract

Today, cardiovascular diseases contribute to approximately 17.9 million deaths annually worldwide. With reference to Europe, coronary artery disease (CAD) causes about 3.9 million deaths annually. Considering the positive physical and psychological outcomes of on-site exercise for CAD patients, this study aimed to expand the literature by examining the effects of a 6-month online exercise training program using wearable devices on CAD patients’ perceived well-being, measured with the PERMA profiler. Individual well-being is considered today as an important prerequisite for healthy societies. Thirty patients with a recent myocardial infarction (i.e., <4 weeks) were randomly assigned to either the online home-based or the community-based exercise group. Both groups followed the same 24-week exercise-based cardiac rehabilitation program three times per week. Each session consisted of a 30-min aerobic, followed by a 15-min strength workout, and then a 15-min balance and flexibility training. The results of the Mann–Whitney U tests and the z scores indicated that the Meaning of Life, Health, Accomplishment, Engagement, and Positive Relationship dimensions of the PERMA were statistically improved, and Negative Emotions were decreased. These findings support the importance of cardiac telerehabilitation for patients’ psychological health, demonstrating that online exercise using wearable devices can be a meaningful alternative to on-site exercise for patients with recent myocardial infarction. These results have policy implications as they provide arguments for providing online exercise for CAD patients as an alternative means for improving their psychological health.

## 1. Introduction

It is well documented today that cardiovascular diseases (CVD) are the leading cause of death globally, contributing to approximately 17.9 million deaths annually [1]. The rise in CVD incidence in developed countries is primarily attributed to lifestyle factors, such as unhealthy eating habits leading to obesity, smoking, increased stress, and lack of physical activity [2,3]. With reference to Greece, according to the latest statistical data (2020), approximately 35% of all deaths were due to CVD, including stroke and coronary artery disease (CAD). Within the Greek population, 8 out of 10 adults possess at least one major modifiable risk factor for CAD, such as diabetes, hypercholesterolemia, hypertension, smoking, and physical inactivity [3,4,5,6]. The overall age-adjusted prevalence of myocardial infarction (MI) in Greece was determined to be 3.6%. Furthermore, it was estimated that approximately 8.7% of all-cause mortality in adults was attributable to physical activity levels below the recommended thresholds [4,5,6].

Regular exercise training is highly recommended for primary and secondary prevention of CAD [7]. Exercise programs for CAD patients should be individualized based on their functional capacity and health status to ensure they are feasible, effective, and safe [8]. Considering that patients with CAD are particularly vulnerable, especially during pandemics like COVID-19, it is advisable to avoid group activities and maintain physical distancing [9]. Recent findings highlight the promise of remotely monitored cardiac rehabilitation (CR) for individuals with heart conditions [5,10]. Recent findings highlight the promise of remotely monitored cardiac rehabilitation (CR) for individuals with heart conditions [5,10]. Remote CR appears to be just as effective as traditional, center-based CR in enhancing cardiovascular risk factors and exercise capacity [11]. Multiple studies have employed wearable sensors, such as heart rate (HR) and electrocardiogram (ECG) monitors, to track the safety and intensity of exercise regimens remotely in people with cardiac conditions [10].

In this study, we aimed to test the effects of a 6-month online exercise training program using wearable devices on CAD patients’ perceived well-being, measured using the PERMA profiler. It is the first time that the effects of such an online exercise program are tested on CAD patients’ perceived well-being, which was measured with a holistic approach given by the PERMA profiler [12]. Well-being is differentiated from indicators of psychological distress, such as depression and anxiety, which may signal poor psychological functioning. In contrast, well-being is characterized by positive feelings, cognitions, and strategies of individuals who function effectively in their lives and appraise it favorably [13]. Seligman [12] advanced a comprehensive conceptualization of well-being through the PERMA profiler, which integrates both hedonic and eudaimonic dimensions, unlike earlier models that focused on only one aspect [14]. Other well-being constructs, such as optimism and emotional vitality, have been linked to cardiovascular outcomes, yet they do not fit neatly within the categories of hedonia or eudaimonia [13]. Well-being is inherently valuable, but it is also linked to advantageous outcomes, including supportive social relationships [15,16], career success [17], and healthier, longer lives [13,18,19].

Based on the above, we hypothesized that online exercise using wearable devices can be a meaningful alternative to on-site exercise for patients with CAD, considering that the greatest barriers seem to be the distance from the rehabilitation center, lack of information about CR, costs, and the fact that some patients perform the exercise at home [11]. A well-organized, safe, and practical cardiac telerehabilitation program is necessary to address the challenges of distance and associated travel expenses [3,4,5]. Implementing appropriate exercise programs at each patient’s home, with real-time monitoring of vital signs (blood pressure, heart rate, oxygen saturation, and body temperature) via modern telemedicine applications, provides a practical solution for effectively and safely exercising patients with chronic diseases [20]. This randomized controlled trial aimed to compare a real-time, home-based cardiac telerehabilitation program—monitored using wearable devices—for individuals with recent MI, to a conventional gym-based CR program, and assess differences in participants’ well-being between the two groups. To our knowledge, this is the first trial to combine telemonitoring via wearable technology with real-time tele-coaching for CR in post-MI patients. 

This study contributes to the literature by testing for the first time the influence of an online exercise training program using wearable devices on CAD patients’ perceived well-being, measured using the PERMA profiler, which provides a holistic approach to the measurement of well-being. It also has policy implications since, if results are positive, it means that such programs can be alternatives to the on-site ones, which are not always suitable for CAD patients due to the risks of trained group programs, which do not provide opportunities for social distancing.

## 2. Literature Review

### 2.1. Remote Exercise and CAD Patients

Recent findings highlight the promise of remotely monitored cardiac rehabilitation (CR) for individuals with heart conditions [5,10]. Remote CR appears to be just as effective as traditional, center-based CR in enhancing cardiovascular risk factors and exercise capacity [11]. Multiple studies have employed wearable sensors, such as heart rate (HR) and electrocardiogram (ECG) monitors, to track the safety and intensity of exercise regimens remotely in people with cardiac conditions [10]. Based on the above, we hypothesized that online exercise using wearable devices could be a meaningful alternative to on-site exercise for patients with CAD, considering that the greatest barriers seem to be the distance from the rehabilitation center, lack of information about CR, costs, and the fact that some patients perform the exercise at home [11]. A well-organized, safe, and practical cardiac telerehabilitation program is necessary to address the challenges of distance and associated travel expenses [3,4,5]. Implementing appropriate exercise programs at each patient’s home, with real-time monitoring of vital signs (blood pressure, heart rate, oxygen saturation, and body temperature) via modern telemedicine applications, provides a practical solution for effectively and safely exercising patients with chronic diseases [20]. This randomized controlled trial aimed to compare a real-time, home-based cardiac telerehabilitation program—monitored using wearable devices—for individuals with recent MI, to a conventional gym-based CR program, and assess differences in participants’ well-being between the two groups. To our knowledge, this is the first trial to combine telemonitoring via wearable technology with real-time tele-coaching for CR in post-MI patients.

### 2.2. Psychological Health and CAD Patients

An expanding body of literature has demonstrated that positive psychological attributes are associated with enhanced health outcomes and increased longevity [21,22], including reduced levels of traditional risk factors for CAD and a lower incidence of CVD [13]. Prospective studies involving patients with pre-existing CAD have revealed that higher levels of positive well-being correlate with a diminished risk of subsequent cardiovascular events and mortality [13,23]. Exercise-based cardiac rehabilitation (CR) is a non-pharmacological, multidisciplinary program that has been found to provide numerous health benefits, such as enhanced cardiorespiratory fitness (CRF), improved quality of life (QoL) and exercise capacity, and decreased both morbidity and mortality rates [24].

While there is a well-established association between psychological distress and increased CVD risk [2], recent research has indicated that well-being independently correlated with CVD. This burgeoning interest has been recognized by the American Heart Association, which highlighted well-being as “an important frontier of knowledge” in its 2030 goal to enhance the health of all population members [25]. Consequently, it is crucial to assess the current evidence linking well-being to CAD. In the Whitehall II study, encompassing nearly 8000 middle-aged British civil servants, participants answered questions about their optimism (i.e., positive expectations for the future), emotional vitality (i.e., active engagement with the world, emotion regulation, and overall sense of well-being), and satisfaction with various life domains (e.g., job, family). Those with moderate to high levels of emotional vitality or optimism had a 20–30% lower risk of incident CAD over five years compared to those with low levels of these traits [26]. Moreover, higher satisfaction across different life domains was linked to a reduced risk of CAD, especially angina [27]. Consistent findings have been observed in nationally representative US samples, where optimism was associated with a lower four-year incidence of heart failure [28], and a sense of purpose in life was connected to a reduced four-year stroke risk among 6800 older adults in the Health and Retirement Study [29]. In the National Health and Nutrition Examination Survey, emotional vitality was found to predict a lower stroke risk over 16 years among more than 6000 participants [30]. Furthermore, positive affect provided a protective effect against the ten-year incidence of CAD in the Canadian Nova Scotia Health Survey [31].

Among patients with existing CVD, higher levels of positive well-being are correlated with a decreased risk of subsequent cardiovascular events and improved health behaviors. Specifically, a greater sense of purpose in life has been associated with a reduced risk of myocardial infarction over a two-year period in individuals with CAD [32]. Moreover, positive effects are linked to a lower risk of myocardial infarction and mortality within two years following percutaneous coronary intervention with stent implantation, while depression and anxiety were not significant predictors of clinical outcomes [33].

### 2.3. Subjective Well-Being and the PERMA Profiler

Subjective well-being is characterized as “the extent to which individuals hold positive evaluations and feelings about their lives as a whole” [34,35]. This concept captures the well-being level experienced by individuals through their comprehensive self-assessment of life satisfaction [36]. Unlike the more objective quality-of-life metrics [37,38], subjective well-being is inherently subjective [36]. As noted by Diener and Ryan [39], an individual’s evaluation of their subjective well-being can include both positive and negative aspects. In the past three decades, empirical surveys on subjective well-being have exploded [40]. Along the same line, extensive research over the decades has established the significance of negative psychological factors—such as depression, anxiety, and hostility—in the onset and progression of cardiovascular disease (CVD) [41,42,43].

Various classifications of well-being have been proposed [18,44,45,46], with the most prominent being hedonic well-being (commonly referred to as happiness, measured through positive emotions and life satisfaction) and eudaimonic well-being (focused on fulfilling one’s potential, assessed through purpose in life).

Seligman [12] advanced a comprehensive conceptualization of well-being through the PERMA profiler, which integrates both hedonic and eudaimonic dimensions, unlike earlier models that focused on only one aspect [14]. Other well-being constructs, such as optimism and emotional vitality, have been linked to cardiovascular outcomes, yet they do not fit neatly within the categories of hedonia or eudaimonia [13]. The PERMA model encompasses distinct key pillars contributing to an individual’s well-being: positive/negative emotions, engagement, positive relationships, meaning, accomplishment, and perceived health. Positive emotions are experienced when individuals feel happiness in their daily lives [47], a state particularly relevant in sports, where recreational activities often foster fun and enjoyment among participants [34,48]. Engagement refers to an individual’s sense of connection, absorption, and involvement in work, leisure activities, or life in general. Highly engaged individuals in intellectual, physical, or psychological leisure activities may achieve a state of ’flow’, characterized by a loss of self-consciousness and total immersion in the activity [49]. Positive relationships involve feelings of sociability, social integration, acceptance, care, and support from others [50]. The accomplishment dimension is particularly relevant in sports contexts, as research shows that even recreational athletes set personal goals (e.g., completing a marathon, improving fitness levels) and seek feedback and positive reinforcement upon achieving these goals [34,51]. The dimension of meaning is notably significant in professional life [52] and in the context of charitable sports events and settings [53]. Finally, the health dimensions refer to individuals’ perceptions of being physically and psychologically healthy.

Following the above discussion this paper aimed to examine the effects of a 6-month online exercise training program using wearable devices on CAD patients’ perceived well-being, measured using the PERMA profiler.

## 3. Materials and Methods

### 3.1. Study Design

This was a single-center, pragmatic, double-blinded (e.g., assessor pre- and post-measurements, independent statistician), two-arm randomized controlled trial conducted in Thessaloniki, Greece. Eligible participants with recent MI (<4 weeks) were recruited from the Cardiology Clinics of the University and private Hospitals of Thessaloniki, Greece, and private physicians’ practices. The exclusion criteria consisted of the following: (1) unstable angina; (2) acute heart failure (HF); (3) severe heart failure with left ventricle ejection fraction (LVEF) < 30%; (4) malignant ventricular arrhythmias; (5) uncontrolled arterial hypertension; (6) musculoskeletal or neurological impairments; and (7) psychological or cognitive disorders that may affect their participation in the exercise programs. All patients provided written consent to participate. The Research Ethics Committee of the School of Physical Education and Sport Science of Thessaloniki (Greece) approved the study protocol, and the study complied with the Declaration of Helsinki. The study has been registered on ClinicalTrials.gov (ID: NCT06071273) [3,4,5].

### 3.2. Patients

After baseline assessment, participants were randomly allocated by an independent statistician into two groups: online home-based (online monitored, home-based exercise group; *n*  =  15) or on-site (in-person attendance at community-based health clubs exercise group; *n*  =  15). The age of the volunteers was coded in nominal categories, as follows: 18–45 years old, 46–55 years old, 56–65 years old, and over 65 years old. Four levels of occupation were included, as follows: private sector, public sector, entrepreneurs, and housewives. Five education groups were included, as follows: secondary education, vocational education, technological education, graduate, postgraduate, and three marital statuses were included, as follows: single, married, and divorced [4,5].

The sample size calculation was based on hypothesized differences in perceived well-being between the exercise and control groups following a 6-month exercise intervention in people with fibromyalgia and age-matched individuals [54]. Using a two-tailed test of significance with a 0.05 two-sided significance level, to achieve a power of 80%, it was estimated that 30 participants in total (i.e., 15 per group) are sufficient for the performed study design. Namely, the study by Gowans [54], was utilized to perform our power sample size calculations. This study used a similar study design assessing the effects of an exercise training program (23 weeks) on mood, depression, anxiety, and perceived well-being in patients with fibromyalgia. Therefore, based on their sample size (e.g., 31 patients in total; 15 EX, 16 CTL), the sample size estimation for our study indicated 27 subjects in total (i.e., for both groups). To compensate for a potential 10% dropout rate, we aimed to recruit at least 30 participants in total for both groups. Similarly, studies in cardiac patients (e.g., Gary et al. [55], Smart [56]) have included sample sizes of no more than 30 participants in total for both groups (i.e., no more than 15 per group). In addition, the long duration (i.e., 6 months) of our exercise intervention, combined with the cardiac condition of our participants (i.e., acute myocardial infarction), which is associated with physical inactivity and poor adherence to exercise programs, makes it challenging to recruit and retain patients. The goal was to recruit at least 30 subjects in total for both groups, assuming a 10% dropout rate.

### 3.3. Well-Being Measurement

The perceived well-being levels were measured with the PERMA profiler [47] on a ten-point Likert scale. This is a valid and reliable measure that has been used in several previous studies. It consists of 18 items categorized into seven dimensions: positive and negative emotions, engagement, positive relationships, meaning, accomplishment, and perceived health. Patients were asked to fill out the questionnaire at baseline and after completing the 24-week program.

### 3.4. Exercise Program Design and Interventions

Both groups followed a similar structure and exercise protocol three times per week for 6 months. Each session consisted of 30 min of aerobic training, approximately 15 min of resistance training, and 15 min of balance and flexibility training. Warm-up (5 min) and cool-down (5 min) periods were also included in the overall exercise session. The exercise intensity was moderate, with a score of 12–14 on the Borg scale. The detailed exercise protocol has been presented in a previous paper [4,5].

The online home-based group underwent a 24-week cardiac rehabilitation program using sensing devices. For our study, Withings’ technology was chosen as the primary remote health monitoring tool. Although various digital monitoring options from companies like Apple, Samsung, and Medopad exist, most of these do not yet offer a complete approach to tracking patients across multiple health dimensions, such as weight, blood pressure, heart rhythm, and sleep patterns [57]. Sensing devices, often equipped with Wi-Fi or Bluetooth capabilities, such as Withing’s Remote Patient Monitoring (RPM) system, are available in both wireless and cellular versions [57]. Withings offers a comprehensive digital medical device toolkit, featuring cloud-based analytics and clinical validation that can be seamlessly integrated into everyday practice [57]. More details are developed below.

### 3.5. Home-Based Group

During the online real-time exercise sessions, all hemodynamic data and readings were continuously transmitted through a mobile application to both the health instructor and cardiologist, who were responsible for delivering and monitoring the session, respectively. These sessions were conducted in real-time using the Zoom (https://zoom.us) platform. In addition to monitoring hemodynamic responses, participants were asked to report their Rate of Perceived Exertion (RPE) at regular intervals throughout the session.

### 3.6. Telemonitoring and Wearable Devices

**Figure 1:**
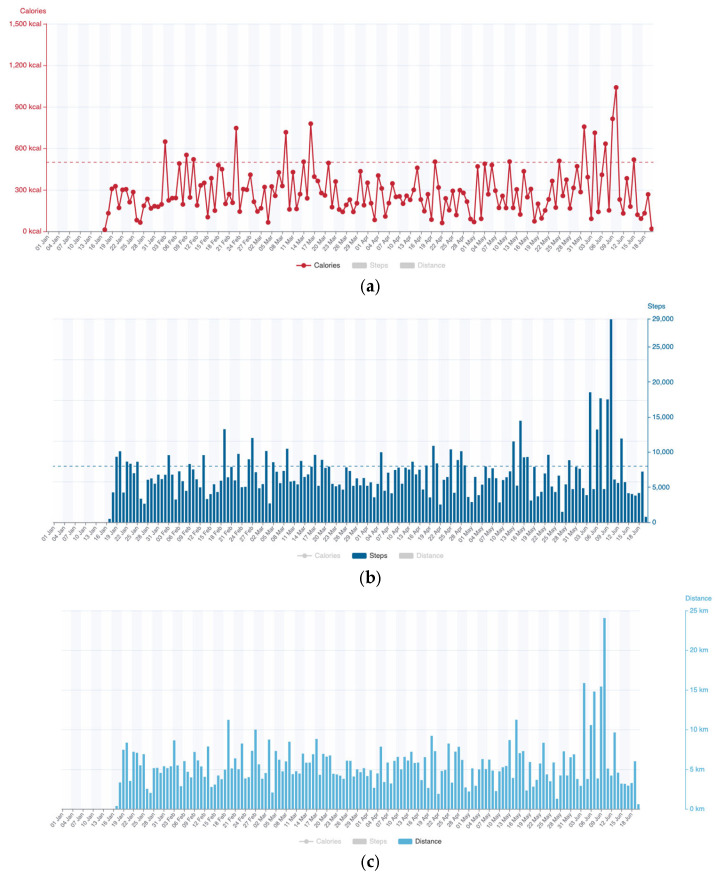
Individualised Scanwatch Report for (**a**) calories, (**b**) steps and (**c**) distance covered during the 6 months.

Participants in the home-based group were provided with the following equipment: (1) a smart watch (Scanwatch; Withings, Issy-les-Moulineaux, France) capable of recording ECG and heart rate (HR); (2) a pulse oximeter for measuring oxygen saturation (SpO2); (3) an automated blood pressure (BP) monitor; and (4) an electric body scale. The Scanwatch is a validated device that allows for 30-s single-lead ECG recordings and performs spot measurements of SpO2. HR is monitored intermittently, while physical activity (e.g., steps) is continuously tracked. The watch incorporates three types of sensors: (1) dry electrodes for ECG, (2) optical sensors for SpO2 and pulse rate, and (3) an accelerometer for physical activity. Additionally, Scanwatch records individual reports of daily calories, steps, and distance covered (see Figure 1). An online platform was developed for cardiologists to access and monitor patients’ hemodynamic responses both during exercise in real-time and at rest.

### 3.7. Gym-Based Group

The gym-based group attended a community health club located near their residences. This group followed the same exercise program as the home-based group but under the supervision of a physical education teacher with experience in cardiac rehabilitation. However, unlike the home-based group, hemodynamic parameters were not monitored during their exercise sessions. The Borg RPE scale was used to assess the patient’s perceived effort and exertion levels. To be included in the analysis, participants from both groups were required to attend at least 80% of the scheduled exercise sessions.

### 3.8. Aerobic Training

The aerobic exercise protocol for both groups involved performing three non-consecutive sessions per week. Each aerobic session, which consisted of an aerobic music workout, lasted 30 min. The intensity of the exercise was set to a moderate level, corresponding to the first ventilatory threshold (VT1), which represents the boundary between light and moderate exercise intensity. For more details, please see our previous study [19]. 

### 3.9. Resistance Training

We designed a resistance training (RT) protocol, similar for both groups, consisting of exercises targeting four major muscle groups (e.g., chest press, low row, lateral arm raises in an upright position, and chair-based bodyweight squats). These exercises were performed using resistance bands (TheraBand, Akron, OH, USA), with 1–3 sets per exercise, 90 s of rest between sets, and 8–10 repetitions per set. The intensity was set at 13–15 on the RPE scale (6–20 points). The total duration of the RT session was approximately 15 min. For more details, please see our previous study [19]. 

### 3.10. Adherence Rates

Adherence to the thrice weekly, 24-week exercise program for the home- and gym-based groups was 91.6% and 90.9%, respectively. The average percentage of peak HR (% HRpeak) for the home-based group was 66.6% ± 4.5 and for the gym-based group was 67.2% ± 5. The average RPE and effect during exercise were, for both groups, 12  ±  1 (“somewhat hard”) and 3  ±  1 (“good”), respectively.

### 3.11. Statistical Analysis

Data analysis was performed using SPSS software version 23 (IBM Corp., Armonk, NY, USA). Frequency counts and percentages were analyzed for categorical data. The Kolmogorov–Smirnov test was used to determine the distribution of data. Mann–Whitney U tests were performed to compare PERMA scores between the different exercise groups (before and after exercise, online and on-site exercise). Statistical significance was set at *p*  ≤  0.05.

## 4. Results

We recruited 15 people (3 women, 12 men) for the online and 15 for the on-site exercise after a recent (i.e., <4 weeks) MI with stable clinical status. The demographic characteristics of the sample are presented in Table 1.

The Kolmogorov–Smirnov test was used to test the normality of the data (involvement, constraint, and motives variables). The sig. value of the test was lower than 0.05 in all the items (0.001 in all of them), showing that the data significantly deviated from normal distribution.

A series of Mann–Whitney U tests subsequently were run and showed the following results:

(a) Online and on-site before exercise: no statistically significant differences were found in the two groups (online and on-site) before starting exercising. This was a requirement to start the intervention.
(b) Online and on-site after exercise. Statistically significant differences were found in Negative Emotions (z = 2.1), Engagement (z = 3.4), and Perceived Health (z = 2.3) (Table 2).
(c) Online before and after exercise. Statistically significant differences were found in all the PERMA dimensions (Table 2).
(d) On-site before and after exercise. Statistically significant differences were found in all the dimensions except Negative Emotions (Table 2). The mean scores of the four groups are also presented in Table 2.

## 5. Discussion

This is the first study to use a holistic measurement of well-being using the PERMA profiler and test if online exercise can help individuals with cardiovascular diseases increase their perceived well-being. The study results are quite encouraging in terms of online exercise using wearable devices. All the PERMA dimensions were shown to improve after the application of the exercise protocol which included 24 weeks of online exercise using wearable devices cardiac rehabilitation program three times per week with aerobic training, resistance training, balance, and flexibility training. As previously noted, research on the effects of online exercise on patients’ psychological health is still limited [1,58,59,60].

The “Perceived Health” was the dimension with the most significant differences before and after online exercise. Previous studies have shown that on-site exercise can have positive outcomes on CAD patients’ physical health [3,4,5,7,61]. Our research extends these findings since this health dimension of the PERMA profiler measures CAD patients’ perception of both physical and psychological health. The exercise protocol that was designed was shown to be a successful program in improving patient’s perceived physical health. Following previous suggestions [3,4,5,61] we used a combination of aerobic training with strength, balance, and flexibility training. The results showed that this program can be delivered even online effectively with the use of wearable devices [3,4,5,61]. It must be noted that, in our study, we measured perceived and not real health, as has been done in some previous studies [11]. It will be interesting to combine these findings with measurements of real physiological measures to examine if real and perceived psychological health are aligned.

The “positive relationship” dimension had the second highest improvement. It involves feelings of sociability, social integration, acceptance, care, and support from others [50]. In the case of online exercise, it involves social integration with other online exercisers and online coaches. The social benefits of exercise are well documented today in healthy populations [62,63,64,65,66,67]. The current study shows that these benefits are also applicable in the case of CAD patients even when they exercise in online settings. It can also be argued that online exercise helps patients to increase their self-esteem, which might help them to socialize more. This has been reported in studies that included healthy populations [34,60] as well as patients [3,11,68].

The “Meaning” pillar was also shown to increase among the patients. The meaning refers to an individual’s feelings that she/he has a meaningful life and adds value to society [52,53]. Online, exercise helped patients to feel that their life is more meaningful as a result probably of their improved psychological state. It is also very encouraging that the Accomplishment pillar was shown to be improved. It refers to the achievement of personal goals, to satisfy the basic human needs of competence, relatedness, and autonomy [69]. It is clear that patients felt that they were successful in completing the exercise protocol and that this gave them a feeling of accomplishment and satisfaction with their exercise goals. Any form of exercise includes a degree of challenge for participants [3], which is further emphasized with the achievement of exercise goals [3]. It is clear that CAD patients perceive exercise as challenging and rewarding at the same time. They pursue the satisfaction of the three main motivation dimensions of competence, relatedness, and autonomy, as given by the self-determination theory [70,71].

The “Engagement” pillar was also shown to have higher scores after the completion of the online exercise protocol. This was not a very expected finding, considering that online classes take place in a home environment and not in a group/social environment. However, exercise patients still had an interaction with the online coaches and the small group of patients who were also online, which might give them a feeling of engagement and belonging to an exercise community. Previous studies among healthy populations have emphasized the value of exercise communities as a main motivation factor for exercise involvement and commitment [34,71,72].

The “Negative emotions” dimension was also improved. This dimension relates mainly to the psychological health of the patients. The results, therefore, showed that online exercise helped patients to reduce their negative emotions. Reduced negative emotions are related to an increase in the sense of fulfillment, strengthening at the same time the sense of a fun and enjoyable life [47]. Both are very important for individuals with cardiac diseases. This is an important finding since previous research has shown that CAD is associated with negative psychological health perceptions for patients, negative emotions, and a lack of willingness to adopt an active lifestyle [3,4,5].

As expected, however, the on-site exercise seems to be more effective in building well-being than the online one in the dimensions of Engagement, Perceived Health, and Negative Emotions. This was an expected finding, considering that on-site exercise creates more opportunities for social interaction, integration, and socialization [3,62]. Therefore, it can be argued that on-site exercise helps individuals to feel more engaged due to the social environment and the social interaction. It is also clear that on-site exercise creates stronger perceptions about the perceived health of the participants, due to the face-to-face contact with the exercise instructors and the trust which is developed. Finally, it contributes more to the reduction in negative feelings because of the social interaction and the environment in which exercise takes place. It is well noted that Accomplishment had similar scores in both types of exercise, which shows that online exercise with the use of wearable devices can help individuals achieve personal goals related to exercise and feel that they have accomplished them. Once again, the protocol that is followed is an important element of the whole process.

Finally, as expected, almost all the dimensions of PERMA were improved among the on-site participants before and after the exercise. This once again provides a strong argument for the value of exercise among individuals with cardiovascular diseases for their psychological health. These results extend our knowledge by showing that not only physiological [21] but also psychological health can be improved by exercising. As previously noted, high levels of well-being are an indicator of healthy communities and societies today [73]. These results, therefore, justify the investment of financial resources from the side of public authorities and government in organizing exercise programs for them. They will increase their quality of life.

## 6. Conclusions

This study showed for the first time that online exercise with the use of wearable devices can have positive effects on the perceived psychological health of CAD patients, and specifically their perceived well-being. All five pillars of the PERMA profiler were improved after the end of the 6-month online exercise program, with the perceived health one, having the highest improvement. The results also showed that while on-site exercise improved perceived well-being more than the online one, there were still dimensions of PERMA, in which the differences were not statistically significant. These results propose that such online exercise programs can be used as an alternative to the on-site ones for CAD patients, in professionals’ effort to convince CAD patients to exercise and create healthy exercise environments for them. Looking ahead, the results of the present study indicate that wider access to affordable health sensing and digital monitoring technologies will enhance personalized care for CVD patients, likely leading to improved quality of life and well-being for patients with recent myocardial infarction.

## 7. Study Limitations, and Suggestions for Future Research

This study is one of the very few that showed with an experimental design that online exercise using wearable devices for individuals with cardiovascular diseases can help them increase their perceived well-being, as measured holistically with the PERMA profiler. However, some limitations should be acknowledged.

First, the results were based on a sample of thirty individuals with cardiovascular diseases. This is a small sample that does not give confidence to generalizations.

However, considering the unique nature of the sample (CAD patients) and the experimental design, it was very difficult to have a larger sample for a 6-month exercise training intervention. These results should, therefore, be confirmed with more studies using larger samples to have confidence in making some generalizations. Studies in other countries with different cultural backgrounds would also be useful for making cross-cultural comparisons.

Due to the small sample, it was also impossible to test any variations in the results related to age and socio-demographic variables. Future studies should control socio-demographic variations such as gender, marital status, education, and socioeconomic status. There might be different perceptions of well-being based on the socio-demographic background of the patients.

A final note should be made about the measurement of well-being. The PERMA profiler is a valid model, as discussed previously, which measures perceived well-being, as has been done in previous studies as well [74,75]. It is, however, a measurement of well-being based on patients’ perceptions. Future studies should correlate the scores in PERMA with real physiological measures taken in a laboratory environment to test if real and perceived health are met. It has to be noted, however, that since well-being is a psychological concept, the use of valid and reliable questionnaires is an acceptable approach. Incorporating the concept of quality of life can also be a proposed line of research for future studies.

## Figures and Tables

**Table 1:** Demographic characteristics of the sample.

Variable		%
Gender	Males	80%
	Females	20%
Age groups	18–45	26.7%
	46–55	20.1%
	56–65	40%
	>65	13.3%
Occupation	Private Sector	13.3%
	Public Sector	26.7%
	Entrepreneur	26.7%
	Housewife	26.7%
Education groups	Secondary Education	6.7%
	Vocational Education	6.7%
	Technological Education	6.7%
	Graduates	66.7%
	Postgraduates	13.3%
Marital Status	Single	13.3%
	Married	66.7%
	Divorced	20.0%

**Table 2:** Mann–Whitney U Tests for examining PERMA differences before and after online and on-site exercise.

Scales PERMA		After Online Exercise	After On-Site Exercise	z Scores	Before Online Exercise	After Online Exercise	z Scores	Before On-Site Exercise	After On-Site Exercise	z Scores
Negative Emotions	Mean Rank	18.9	12.0	−2.1 *	11.4	19.5	−2.5 **	13.1	17.8	−1.4, n.s.
	Sum of Ranks	284.5	180.5		171.5	293.5		197.5	267.5	
	Mean Score	5.62	4.62		4.26	5.62		3.93	4.62	
Positive Relationships	Mean Rank	13.9	17.1	−1.0, n.s.	10.8	20.3	−2.9 **	11.7	19.3	−2.4 **
	Sum of Ranks	208.5	256.5		163.0	302.0		175.5	289.5	
	Mean Score	8.82	8.97		7.28	8.82		8.26	8.97	
Engagement	Mean Rank	10.0	20.9	−3.4 ***	11.8	19.2	−2.3 *	8.9	22.0	−4.1 ***
	Sum of Ranks	151.0	314.0		177.0	288.0		134.5	330.5	
	Mean Score	8.08	9.02		7.28	8.08		7.57	9.02	
Perceived Health	Mean Rank	11.8	19.2	−2.3 **	10.2	20.8	−3.3 ***	8.2	22.7	−4.5 ***
	Sum of Ranks	177.0	288.0		153.0	312.0		124.0	341.0	
	Mean Score	8.22	8.71		6.53	8.22		6.84	8.71	
Meaning of life	Mean Rank	12.7	18.2	−1.8, n.s.	12.1	18.8	−2.1 *	11.2	19.8	−2.8 ***
	Sum of Ranks	191.5	273.5		182.5	282.5		168.0	297.0	
	Mean Score	8.46	8.76		7.63	8.46		7.83	8.76	
Accomplishment	Mean Rank	16.0	15.0	−0.31, n.s.	11.6	19.3	−2.4 **	10.6	20.3	−3.0 **
	Sum of Ranks	240.0	225.0		175.0	290.0		160.0	305.0	
	Mean Score	8.17	8.24		7.51	8.17		7.48	8.24	
Positive Emotions	Mean Rank	14.1	16.8	−0.86, n.s.	12.4	18.5	−1.9 *	12.1	18.9	−2.1 *
	Sum of Ranks	212.0	253.0		187.0	278.0		181.5	283.5	
	Mean Score	8.15	8.37		7.48	8.15		7.73	8.37	

* *p* < 0.5, ** *p* < 0.01, *** *p* < 0.001. n.s.: non significand

## Data Availability

We do not prefer to share our raw data publicly due to the nature of the sample (we did not ask for this in the consent form).
